# Perceived Barriers and Facilitators to Physical Activity in Patients Undergoing Bariatric Surgery: A Systematic Review of Qualitative Studies

**DOI:** 10.1007/s11695-026-08735-w

**Published:** 2026-05-21

**Authors:** Veronika Szabóová, Klára Daďová, Tomas Vetrovsky, Manuel González Sánchez, Anna Vacová, Martin Matoulek

**Affiliations:** 1 Faculty of Physical Education and Sport, Charles University, Prague, Czech Republic; 2 Department of Physiotherapy, University of Malaga, Málaga, Spain; 3 3rd Internal Clinic, First Faculty of Medicine and General University Hospital in Prague, Charles University, Prague, Czech Republic

**Keywords:** Obstacles, Motivators, Exercise, Metabolic bariatric surgery

## Abstract

People with obesity encounter barriers that shape adherence to lifestyle interventions. This systematic review examined barriers and facilitators to physical activity from the perspective of patients undergoing bariatric surgery. A literature search across 4 databases identified studies published through July 31, 2025; 16 met inclusion criteria. 4 overarching thematic units emerged: internal psychological, internal physical, external social, and external environmental factors. Internal physical barriers were the most frequently reported (14 studies), with pain and functional limitations being the most common. Among facilitators, internal psychological factors predominated and were cited in 11 studies. Behavioral strategies and positive associations with physical activity were the most frequent. Overall, the findings highlight physical and psychosocial determinants influencing engagement in physical activity among this population and support multilevel interventions to enhance participation.

**Supplementary Information:**

The online version contains supplementary material available at 10.1007/s11695-026-08735-w.

## Key points

16 studies identified 4 key thematic units influencing physical activity in patients undergoing bariatric surgery: internal psychological, internal physical, external social, and external environmental factors.

Internal physical factors, particularly pain and functional limitations, were the most frequently reported barriers.

Internal psychological factors, including behavioral strategies and positive associations with physical activity, were the most common facilitators.

**Supplementary Information:**

The online version contains supplementary material available at 10.1007/s11695-026-08735-w.

## Introduction

Obesity is recognized as a chronic, relapsing, and multifactorial disease by major health organizations, including the World Health Organization, the Obesity Medicine Association, and the American Medical Association [1–3]. From 1990 to 2022, the percentage of adults aged 18 and over with obesity more than doubled from 7% to 16% [4].

Metabolic bariatric surgery (MBS) is currently the most effective treatment modality for severe obesity and associated metabolic diseases [5]. Long-term studies published after 1991 have consistently demonstrated the greater effectiveness of surgical procedures in reducing body weight compared to non-surgical approaches [6–8].

Physical activity (PA) and exercise training are integral components of comprehensive care for patients undergoing bariatric surgery and should be incorporated into both preoperative and postoperative care regimens [9–12].

One of the key factors for maintaining weight loss over time is long-term adherence to PA. However, for patients undergoing MBS, this is often complicated by musculoskeletal problems and coexisting chronic conditions that limit exercise tolerance and adherence [13]. The extant literature indicates that most patients remain insufficiently active after MBS [11, 14, 15]. Barriers to PA are also commonly reported in candidates for MBS [16].

In order to ensure the effective planning and integration of PA into the daily lives of patients undergoing bariatric surgery, healthcare professionals and patients must address the multifactorial determinants influencing PA levels. Specifically, studies identify perceived challenges related to self-discipline and time constraints as primary barriers to physical activity among individuals living with obesity. Conversely, social support and motivation for improvement are recognized as significant facilitators of PA [17]. Furthermore, the literature suggests that gender differences [18] and perceptions of environmental safety [19] necessitate an individualised approach to interventions. Understanding these determinants is essential for developing biopsychosocially informed interventions that help individuals living with obesity overcome internalised barriers such as fear of pain [20] or social stigma [21].

Although individual studies addressing barriers and facilitators of PA in candidates for MBS and patients after surgery provide valuable insights, the available literature lacks a systematic overview that would comprehensively integrate these findings. The absence of such an overview significantly limits the possibilities for the transferability of the findings and their effective application across different clinical contexts. Given that many of the determinants are subjective, context-dependent, and embedded in lived experience, they may not be fully captured by quantitative approaches. Qualitative research addresses this gap by offering in-depth insight into how individuals perceive and navigate barriers and facilitators to physical activity, supporting the development of tailored interventions.

This systematic review therefore aims to investigate the qualitative literature exploring barriers and facilitators to PA from the perspective of patients undergoing bariatric surgery and to systematically synthesize its findings.

## Methods

The systematic review was conducted in accordance with PRISMA 2020 [22]. The review protocol was registered in PROSPERO (ID: CRD42024439572).

### Eligibility Criteria

The review included qualitative studies examining factors influencing participation in PA among individuals who were candidates for, or had undergone, MBS. There were no restrictions on age or gender. Case reports, quantitative studies, non–peer-reviewed publications, non-English articles, and abstracts or conference proceedings were excluded. Full inclusion and exclusion criteria are provided in Table S1. The criteria were defined during PROSPERO registration and redefined after a pilot search. The search was limited to studies published up to July 31st, 2025.

To define PA, the most widely used definition among researchers was that of Caspersen et al., who defined it as any bodily movement induced by skeletal muscles that results in energy expenditure [23]. The barrier was defined in accordance with the Oxford Dictionary as a circumstance or obstacle that hinders communication or progress, while the facilitator was defined as a person or factor that enables something [24].

### Search and Screening

The search was conducted in four databases: PubMed, Web of Science, EBSCO and Scopus. The search formula was derived from predefined keywords related to bariatric surgery, barriers and facilitators, physical activity and exercise, and was refined based on a pilot search. The search formula included free-form text, wildcards, Boolean operators and combinations of MeSH terms specific to individual databases. Full search formulas for each database are provided in Table S 2. The final search was performed on August 26th, 2025.

All the articles that were found were merged into a single database using the Rayyan tool [25], where duplicates were automatically identified and excluded from the selection process. The screening comprised of three phases. The initial screening was based on the title, followed by an assessment of the abstracts and, in the final stage, the full texts of the studies. Each of these three phases was carried out independently by two researchers, and then verified. In the event of disagreement, a meeting was convened to reach a consensus. If a disagreement could not be solved, a third reviewer was consulted.

### Quality and Risk of Bias Assessment

The quality of the included studies was assessed using the CASP Checklist For Qualitative Research [26], a tool recommended by the Cochrane for systematic reviews of qualitative studies [27]. If a study received a “yes” response less than 8 times, it was deemed insufficiently high quality to be included in the review.

### Data Extraction, Analysis and Synthesis

The thematic synthesis method according to Thomas and Harden [28] was used for data extraction, analysis and synthesis and the process was carried out in several phases. First, the researchers familiarized themselves with all 16 publications included to the review. During this phase, the studies were read repeatedly so the researchers could gain insight into each publication, as well as their similarities and differences. The familiarization phase was followed by the data extraction phase. A relevant qualitative data was first systematically extracted from the results and discussions of the included studies and organised into an extraction table. If a given study had already performed data coding or defined topics, these topics were entered into the extraction table. Otherwise, an inductive approach was used to mark relevant sections of text with descriptive codes. Uncodified participant quotes or text passages containing descriptions of phenomena, or author interpretations were read repeatedly to gain an in-depth understanding of the context and create initial descriptive codes which were entered into the extraction table. To preserve the fidelity of the original data, coding was performed without predefined categories. The data extraction and coding were conducted independently by two researchers and then verified. In the event of a disagreement, a meeting was held to reach a consensus. The intitial codes were then grouped into descriptive themes, analythic sub-themes, analythic themes and thematic units. These were critically reviewed in the context of the entire dataset, with redundant codes or sub-themes eliminated. Finally, the two researchers discussed the results of the thematic analysis to reach an agreement on the coding scheme, as well as the emerging thematic units, themes and sub-themes.

## Results

**Fig. 1:**
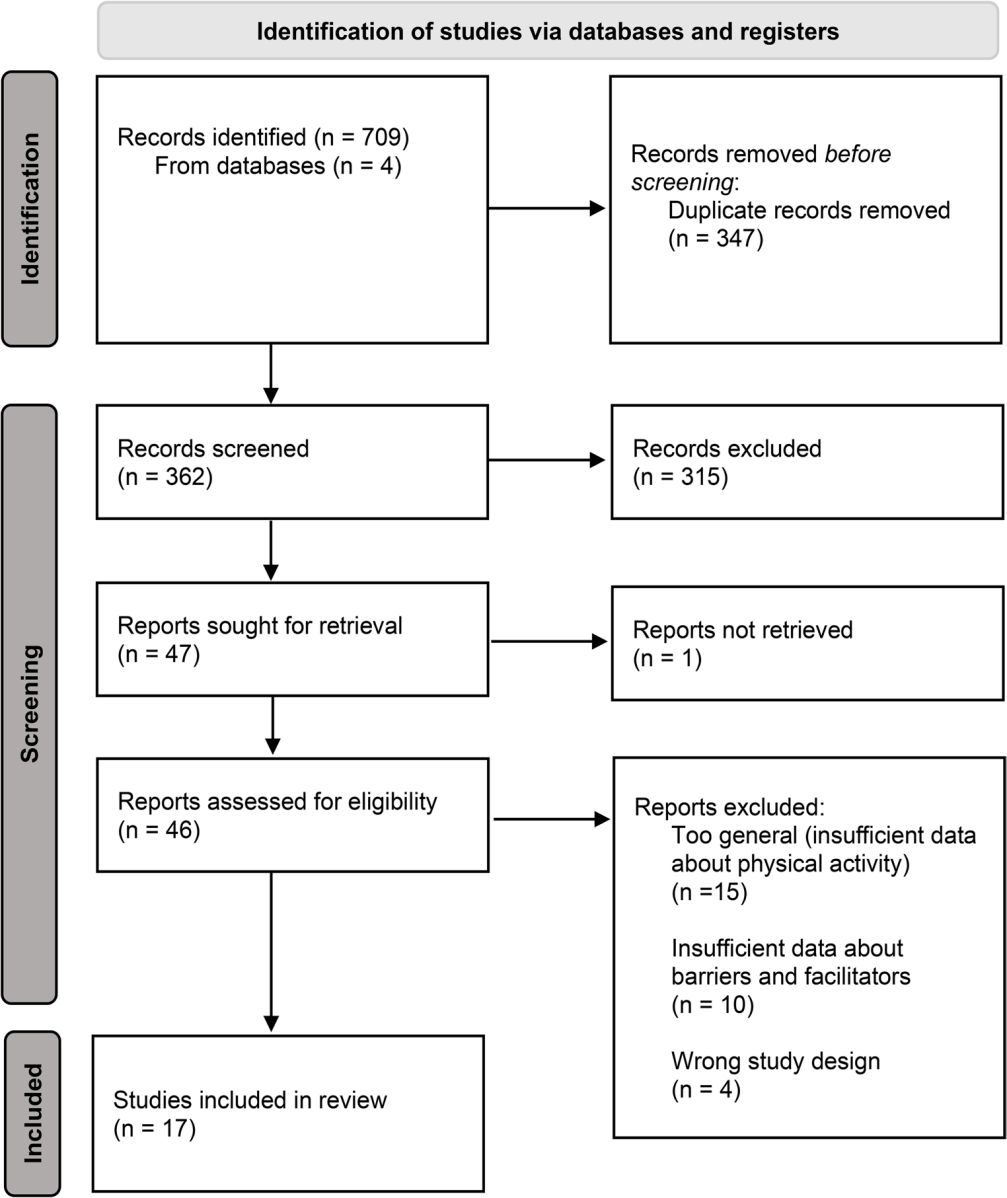
PRISMA Flow diagram (Page et al., 2021)

To ensure transparency of the review, the PRISMA 2020 (Preferred Reporting Items for Systematic Reviews and Meta-Analyses) scheme shown in Fig. 1 was used to document the decision-making process for assessing the eligibility of studies.

### Study Characteristics

Out of 17 studies which have met the eligibility criteria, 1 study was excluded in the manner of insufficient quality. The results of the quality assessment and risk of bias for the included studies are recorded in Table S 3. A total of 16 studies [29–44] published between 2010 and 2025 were analysed, with a total of 621 participants. In terms of gender distribution, women were significantly overrepresented, accounting for 89% (*N* = 550) of the sample, while men accounted for 11% (*N* = 71). The median sample size across the studies was 14.5, ranging from 6 to 366 participants. The majority of studies collected data only after MBS. Specific data are provided in Table 1. 

**Table 1:**
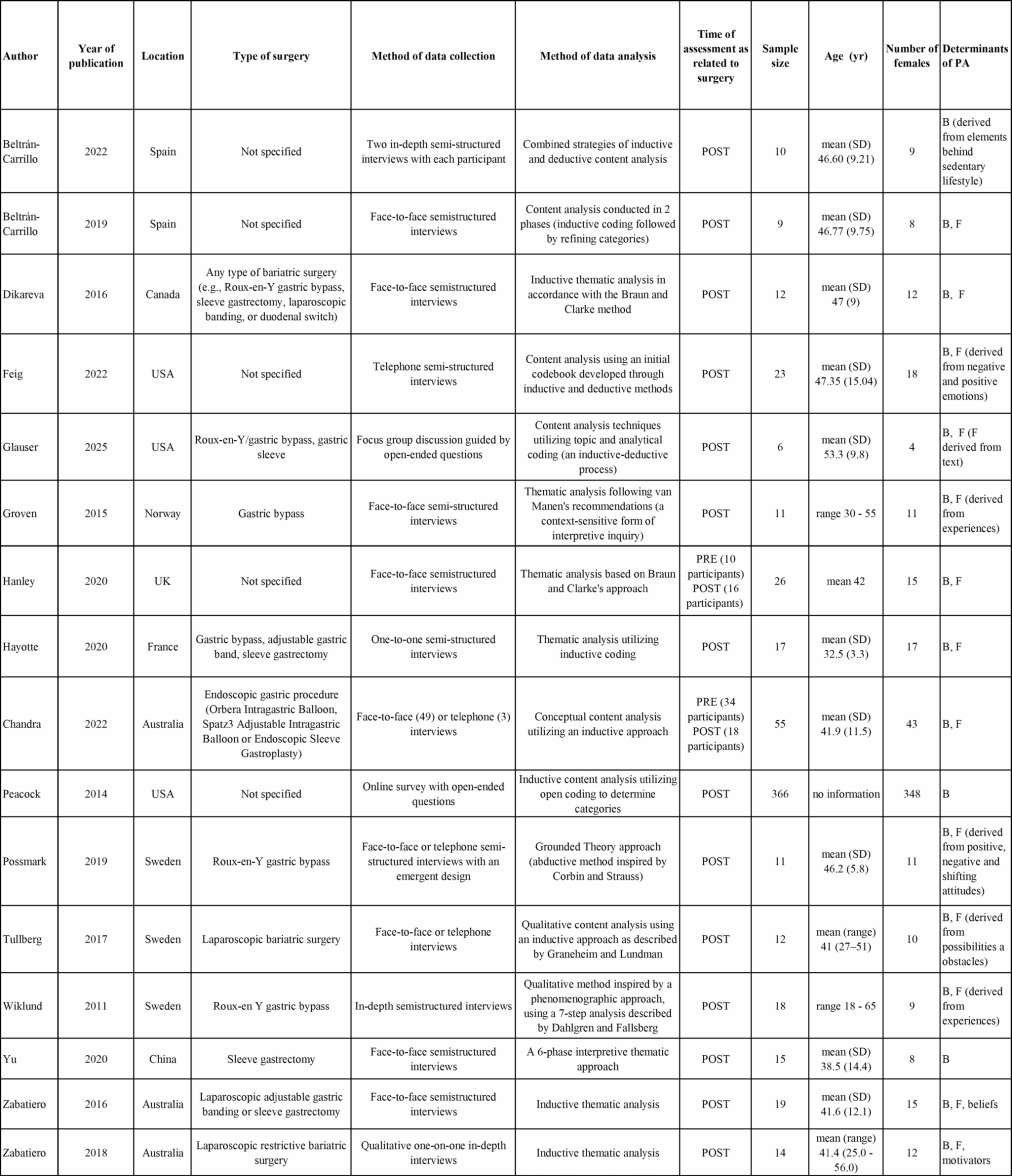
Characteristics of analyzed studies

**Fig. 2:**
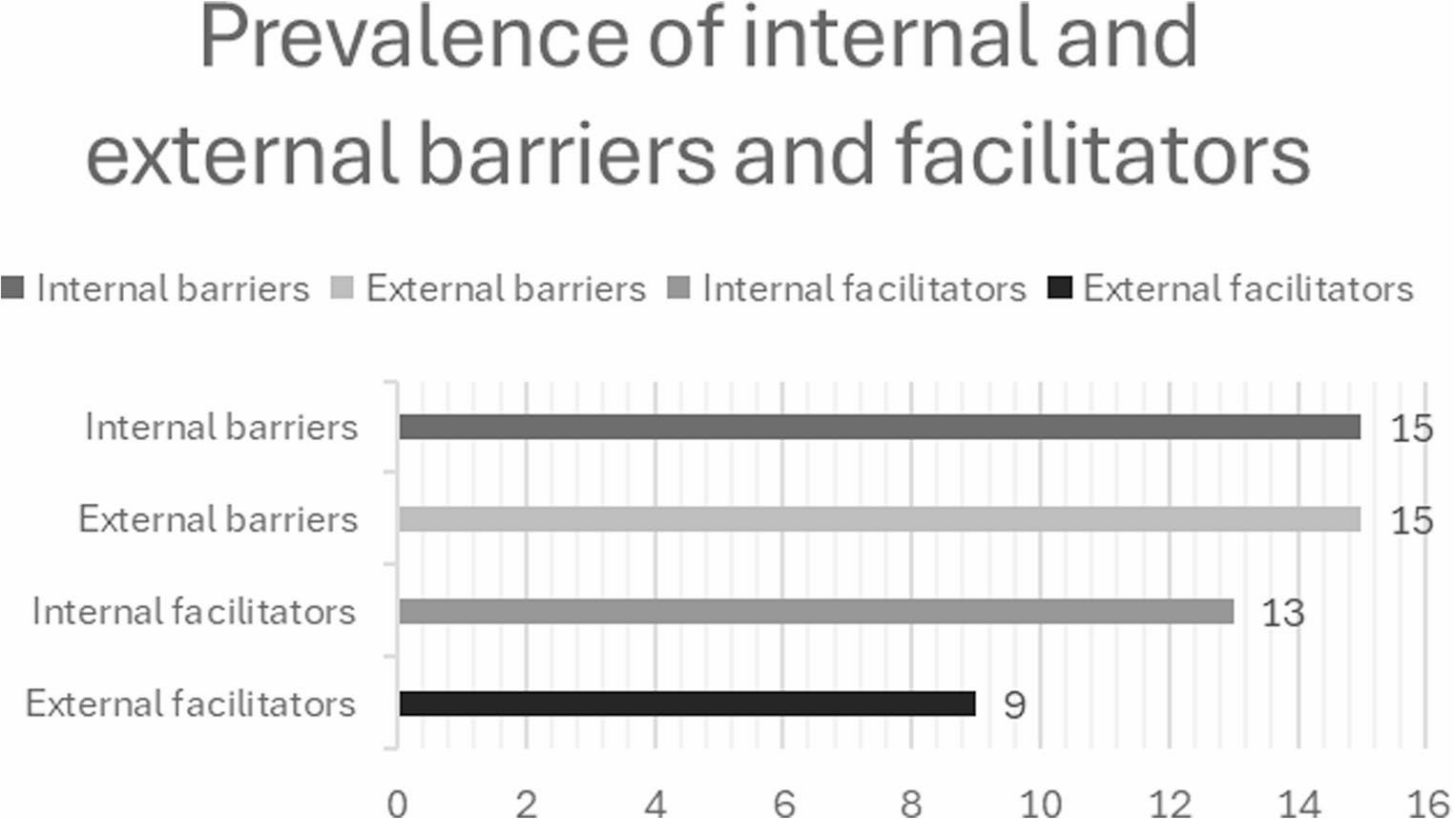
Prevalence of internal and external barriers and facilitators

Of the 16 studies analysed, all have addressed barriers and 13 identified facilitators of PA. Those barriers and facilitators can be divided into internal and external (for their representation see Fig. 2). Through the thematic synthesis, we grouped identified barriers and facilitators into 4 thematic units, analytical themes and sub-themes.

The internal barriers and facilitators can be further subdivided into psychological and physical thematic units. The external barriers and facilitators can be further subdivided into social and environmental thematic units. The thematic units of barriers and facilitators in individual publications are delineated in Table 2. The subsequent subchapters present the individual thematic units of barriers and facilitators, along with their specific analytical themes and sub-themes.

**Table 2:**
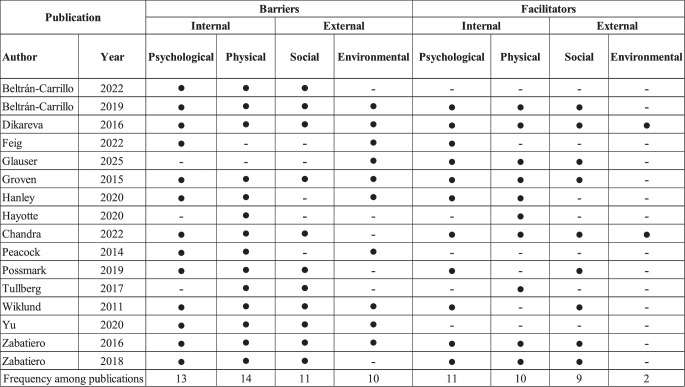
Thematic units of barriers and facilitators

*"•"* identified, *"-"* not identified

### Barriers

The most prevalent barriers identified across the studies were internal barriers - physical (14 studies) and psychological (13 studies). Social barriers were identified in 11 publications while environmental barriers were the least frequently represented.

**Fig. 3:**
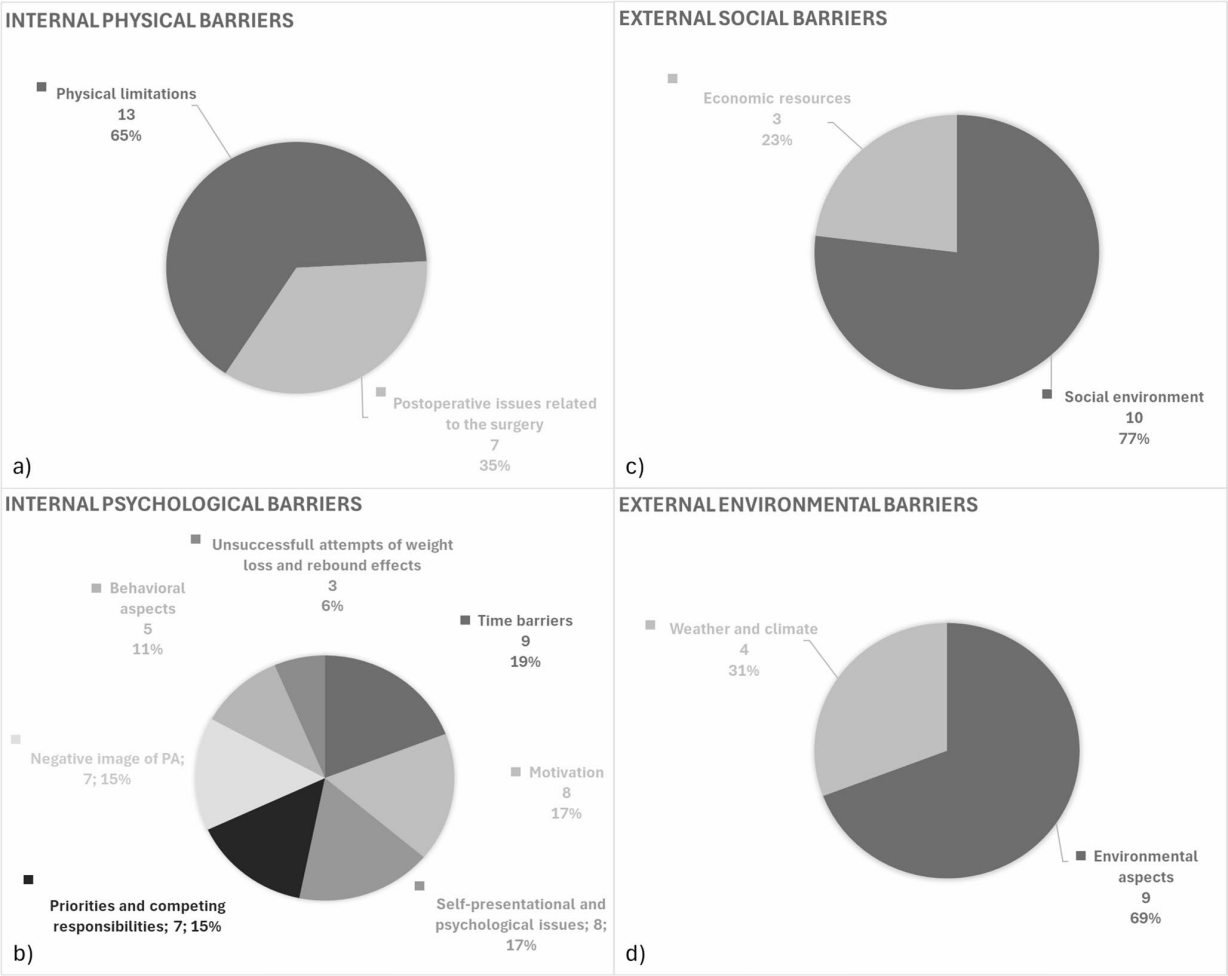
Barriers to PA - visualisation of analytical themes. **a**) Internal physical barriers; **b**) Internal psychological barriers; **c**) External social barriers; **d**) External environmental barriers

The following section details the analytical themes within individual thematic units, which are also visualised in Fig. 3. A comprehensive inventory of the analytical sub-themes and descriptive themes concerning the barriers that were identified in the analysed studies can be found in the supplementary materials, specifically in Table S4.

#### Internal Physical Barriers

The most frequently mentioned analytical theme was “physical limitations” (13 studies). Within this theme, barriers in the form of fatigue and lack of energy, pain, reduced physical fitness, mobility, and health problems were repeatedly specified. The theme “postoperative issues related to the surgery” was identified in 7 studies, with the most frequently mentioned barrier being problems associated with excess skin.

#### Internal Psychological Barriers

The most frequently identified internal psychological barriers fell under the theme of “time barriers”. Barriers related to the theme of “motivation” and “self-presentational and psychological issues” were also commonly reported, each identified in 8 studies. Themes related to “negative image of PA” and “priorities and competing responsibilities” were reported with equal frequency, each appearing in 7 studies. Most commonly identified barriers falling under the theme of “behavioral aspects” were different preferences for leisure activities and insufficient organizational skills.

#### External Social Barriers

A total of 10 publications identified the analytical theme of “social environment.” This finding emerged as the second most prevalent analytical theme in terms of both barriers and facilitators, with the primary barrier identified as inadequate social support. In contrast, barriers falling under the theme of “economic resources” were represented significantly less frequently.

#### External Environmental Barriers

The theme “environmental aspects” was identified in 9 publications, and a recurring barrier was “limited access to specialised exercise facilities or equipment”. Barriers classified under the theme “weather and climate” were mentioned in 4 studies.

### Facilitators

Facilitators were represented in 13 of the publications analysed with internal facilitators being described in all studies, while external ones only in 9 of them.

As illustrated in Table 2, a mere 2 publications identified facilitators from all 4 thematic units, namely psychological, physical, social, and environmental facilitators.

The predominant thematic unit of facilitators identified across the studies examined were psychological facilitators. The thematic analysis identified physical facilitators in 10 publications, and social facilitators in 9 publications. The role of environmental facilitators was represented on only a limited basis.

**Fig. 4:**
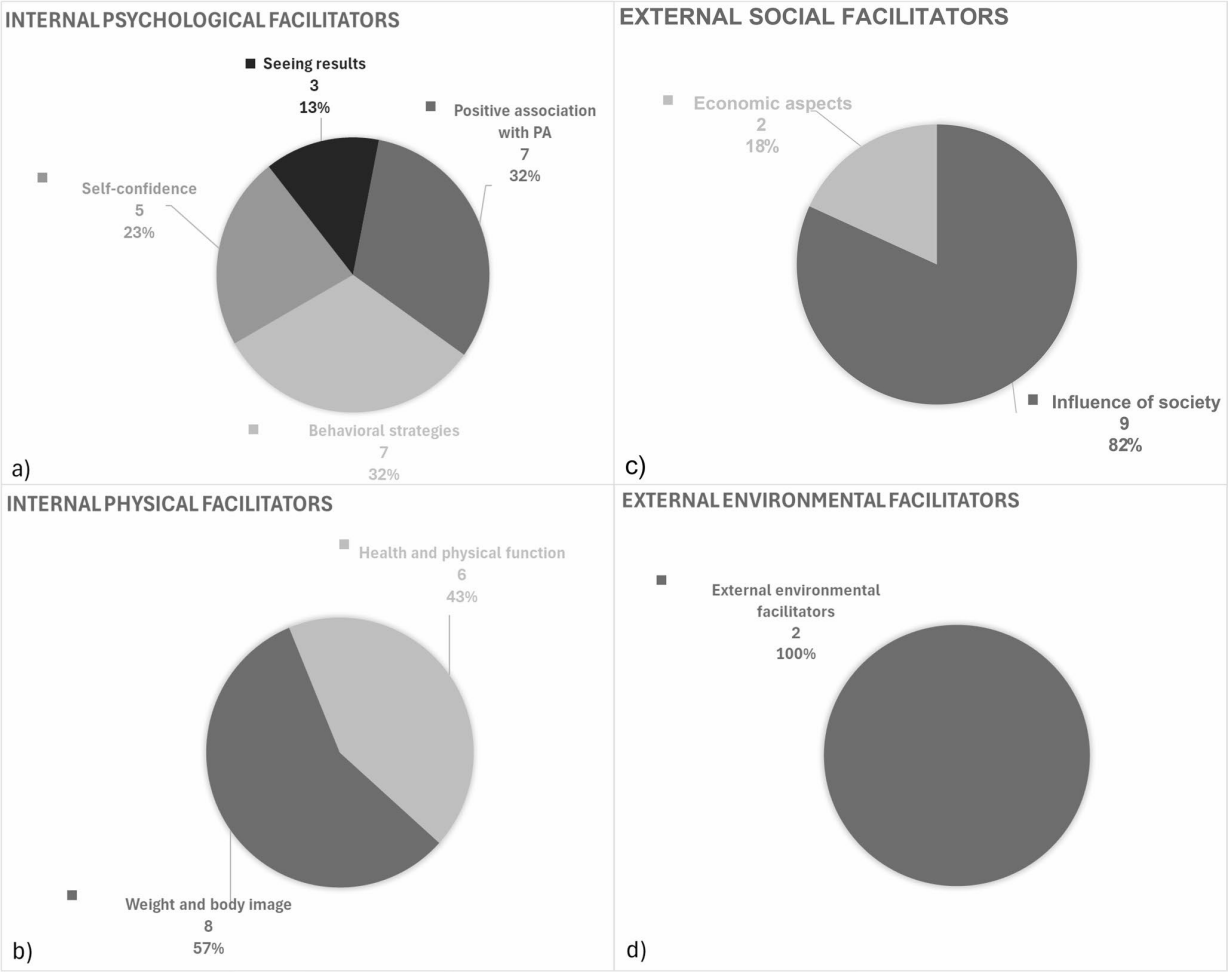
Facilitators to PA - visualisation of analytical themes. **a**) Internal psychological facilitators; **b**) Internal physical facilitators; **c**) External social facilitators; **d**) External environmental facilitators

The following section as well as Fig. 4 details the analytical themes identified within each thematic unit. For detailed enumeration of the analytical sub-themes and descriptive themes see supplementary materials, Table S 5.

#### Internal Psychological Facilitators

Across all studies, the following 4 analytical themes of psychological facilitators were identified: theme of “positive associations with PA” mostly involved positive experiences from PA such as improving mood; theme of “behavioral strategies,” which also incorporates the concepts of improved time management and planning; theme of “self-confidence” and theme of “seeing results”.

#### Internal Physical Facilitators

The identified internal physical facilitators could be divided into analytical themes: “weight and body image” and “health and physical function”. The facilitator of weight loss appeared in individual studies in relation to the future, as weight loss as a result of PA or weight stabilization as a result of PA, but also as weight loss already achieved as a result of surgery, which facilitated subsequent participation in PA.

#### External Social Facilitators

The analysis of the extant literature revealed 2 analytical themes of external social facilitators. The theme “influence of society” emerged as a facilitator in a total of 9 studies. The most prevalent social facilitator was social support, which was found to increase motivation to engage in PA when exercising in a group. Conversely, the theme “economic aspects” was identified in a mere 2 studies.

#### External Environmental Facilitators

Only two studies identified external environmental facilitators, including the availability of appropriate facilities or equipment, access to health information and exercise-related knowledge.

## Discussion

This systematic reveiew of qualitative studies aimed to investigate barriers and facilitators to PA from the perspective of patients undergoing bariatric surgery. The findings suggest that both internal and external factors influence PA in this population. The thematic synthesis of the 16 included studies identified 4 overarching thematic units: internal psychological, internal physical, external social, and external environmental factors. These findings correspond with recent systematic reviews in the general population [45, 46] and among individuals living with obesity [15], which also emphasize the interaction of individual and environmental determinants.

When interpreting the findings, it is essential to note that qualitative data capture the subjective, lived experiences of the patients. Therefore, the prominence of certain internal and external factors reflects their salience in participants’ narratives rather than a quantitatively measurable effect. The interpretation of results must be grounded in this understanding. Importantly, the interpretation of these findings should be situated within the characteristics of the included study populations. Most studies predominantly involved female participants and focused on the postoperative period, suggesting that the identified barriers and facilitators may primarily reflect gendered experiences of physical activity and postoperative perceptions and experiences related to engaging in physical activity.

### The Prominence of Internal Factors in Patient Narratives

The findings suggest that internal barriers, particularly physical limitations such as pain and fatigue, and psychological factors including low motivation and time constraints, are more frequently described as salient than external barriers. This pattern is consistent with findings reported in previous research involving individuals with obesity, where psychological and physical limitations similarly predominate over environmental or social factors [17].

Psychological barriers to PA in population of patients undergoing bariatric surgery often originate from long-standing experiences with obesity, repeated unsuccessful weight-loss attempts, and chronic exposure to stigma. These factors contribute to low self-confidence, negative body image, and shame when exercising in public. Maladaptive coping patterns, including emotional overeating in response to stress or trauma, further shape attitudes toward PA [30, 35]. In addition, the perception that PA is worthwhile only when performed at a certain level of intensity or duration may also discourage engagement, as individuals often withdraw from activity when they feel unable to meet these expectations [32].

The frequent reporting of internal over external factors was even more pronounced for facilitators, which were consistently described by participants as being internally driven. A notable finding concerns differences in motivation types across stages of the postoperative trajectory. While external motivators, such as rapid weight loss and visible bodily changes, play an important initiating role shortly after surgery [37], long-term adherence depends on the gradual development of intrinsic motivation. Feig et al. [32] describe how experiences of mastery and competence, such as lifting heavier weights or improving endurance, contribute to the emergence of intrinsic motivation. These performance-related achievements encourage patients to derive satisfaction and enjoyment from PA itself rather than viewing it solely as a weight-loss tool.

Furthermore, the prominence of internal psychological and physical determinants may partly reflect the methodological orientation of the primary qualitative studies. In-depth interviews and focus groups inherently aim to generate a subjective understanding of how people perceive, reflect, and interpret their experiences [47].

### The Ambivalent Role of Social Factors

External determinants play a complex and often ambivalent role. Social support serves as a significant facilitator when present, but its absence constitutes a major barrier. At this level, the key facilitators are a supportive family and therapeutic context, as well as opportunities for social interaction and participation in PA. This finding is consistent with, for example, review examining the general population [46]. Studies indicate that support is not merely passive presence, but requires active engagement from family and friends – for example, participating in exercise together or assisting with daily responsibilities [29, 39, 43]. Conversely, well-intentioned support can be perceived as intrusive oversight or “nagging,” which individuals living with obesity often experience negatively and may lead to resistance [30, 33].

Furthermore, stigmatization within the exercise environment also represents a substantial barrier. Individuals living with obesity frequently report feelings of being “different,” “clumsy,” and “observed” in commercial gyms, which they perceive as non-inclusive spaces where they are marginalized and staff do not pay attention to them [30, 41]. For some individuals living with obesity, gym membership and sports equipment further impose significant financial burdens [29, 43].

### Environmental Barriers vs. Environmental Facilitators

A notheworthy finding is that while environmental barriers were extensively documented in the analysed publications (9/15), environmental facilitators were identified in only 2 studies. This may reflect the true limitations of the environment, but it could also be indicative of a tendency to perceive barriers rather than facilitators. In addition, this pattern may be influenced by the qualitative methodological approach: rather than objectively mapping external conditions, interviews and focus groups encourage participants to engage in self-narration and subjective sense-making [47]. This methodological tendency to center on the individual’s inner perspective may naturally limit the identification of environmental facilitators.

*Temporal shifts*.

Our research, although predominantly based on postoperative studies, suggests that the perception of barriers shifts dynamically over time. Prior to surgery, physical barriers associated with excess weight (such as pain, dyspnea, clumsiness, and pervasive fatigue) are the most dominant. Patients often perceive their bodies as obstacles and view weight loss as a prerequisite for engaging in PA [41]. Following surgery, a substantial transition occurs. With rapid weight loss, physical barriers diminish and patients report improved tolerance for movement, which becomes a critical facilitator. However, new, surgery-specific barriers emerge, such as excess skin, which can physically impede activity and simultaneously serve as a new source of body dissatisfaction and shame [29, 31, 32, 40].

Additionally, shifts in the perception of priorities can be observed, with patients following MBS indicating the prioritisation of their own needs and placing themselves and their health at the pinnacle of their list of priorities [34].

Over the long term, as demonstrated by Hanley et al. [35] and Hayotte et al. [36], initial enthusiasm may dissipate, and disappointment related to weight plateaus or recurrent weight gain may emerge, leading to feelings of failure and relapse into a sedentary lifestyle.

### Implications and Future Directions

A multifaceted biopsychosocial approach is essential for effectively integrating PA into the lives of patients undergoing bariatric surgery. Interventions should incorporate behavioral strategies, motivational interviewing, cognitive-behavioral techniques targeting maladaptive thought patterns, and the development of adaptive coping skills. Standardized questionnaires that monitor patients’ barriers and facilitators to PA would enable more consistent and comparable assessments. Group-based activities and family education can strengthen social support; however, families should be trained to provide encouragement that is constructive rather than controlling. Offering activity options that minimize discomfort and promote a sense of mastery, such as aquatic exercise, may further enhance long-term adherence. Additionally, improving access to specialized PA programs, including telemedicine and online formats, remains crucial.

Professional support must be personalized, continuous, and aligned with the patient’s current stage of treatment. Preoperative preparation plays a critical role in promoting postoperative PA adherence and should prioritize addressing psychological barriers and fostering realistic expectations. In the early postoperative stage, education about physical limitations and management of bodily changes, combined with the reinforcement of initial successes, supports externally driven motivation. Long-term efforts, however, should center on relapse prevention and cultivating intrinsic motivation. Interventions that emphasize positive exercise experiences and highlight improvements in health and well-being may facilitate the shift from externally driven engagement to sustainable, intrinsically motivated PA participation.

Future research should place greater emphasis on male population and the preoperative period. Findings from broader populations with obesity suggests gender-specific differences in perceived barriers, with men more frequently reporting physical limitations and women more often identifying psychosocial barriers [18], underscoring the need for gender-sensitive interventions.

Longitudinal research tracking PA determinants over multiple years is also required to better understand how these factors evolve over time and to clarify the dynamics underlying changes in motivation, barriers, and facilitators.

### Strengths and Limitations

This is the first comprehensive synthesis of qualitative research on barriers and facilitators to PA among patients undergoing bariatric surgery, and its findings provide a foundation for the development of targeted clinical recommendations.

Conceptually, a major strength of the review is its ability to capture subjective, context-dependent determinants of PA embedded in lived experiences, which are frequently overlooked by standardized quantitative methodologies. Another strength of this review is the geographical and cultural diversity of the included primary studies. The synthesized data originate from a range of environments and healthcare systems across Europe, North America, Australia, and Asia, allowing for the identification of patterns in how barriers and facilitators to physical activity are experienced across different contexts. This diversity provides a broader basis for considering the potential transferability of the findings to other settings.

The review exhibits several limitations, largely reflecting the constraints of the underlying primary literature. A primary limitation is the significant gender imbalance within the synthesized sample. Although this disparity aligns with the epidemiological demographics of the population of patients undergoing bariatric surgery, it likely shapes the synthesis by emphasizing barriers and facilitators that are more salient in women’s experiences, thereby limiting the transferability of findings to male patients.

Furthermore, there is a distinct temporal bias in the analyzed literature. The findings of this review are strongly shaped by the predominance of studies focusing on the postoperative period. The vast majority of the 16 included studies focused exclusively on the postoperative period, with preoperative data obtained in only 2 instances. As a result, the identified barriers and facilitators largely reflect experiences associated with post-surgical recovery, rapid weight loss, and adaptation to bodily changes, rather than preoperative expectations or long-term trajectories. The review highlights a critical lack of longitudinal qualitative research, which restricts the ability to fully understand how perceived determinants dynamically evolve over multiple years post-surgery.

The methodological approaches used across the included studies also influence the synthesis and its interpretation, serving as both a strength and a potential limitation for this review. The included studies demonstrate methodological heterogeneity; while many relied on purely inductive coding, several utilized combined inductive-deductive strategies, and others applied specific interpretive frameworks such as Grounded Theory or phenomenography. While this diversity provides a rich, multi-faceted pool of data, it also implies that some of the primary findings were subtly shaped by specific theoretical or abductive lenses prior to our synthesis. Therefore, the aggregated thematic units represent a complex blend of participant accounts and interpretations generated through different analytical frameworks.

Additionally, the thematic synthesis itself is shaped by the methodological application of Thomas and Harden’s approach [28]. While this inductive method allowed for the generation of analytically developed, overarching themes, the aggregation of qualitative data across heterogeneous studies necessitates a level of abstraction. Thus, while the synthesis provides a broader basis for considering the transferability of the identified barriers and facilitators, it may obscure context-specific nuances present in the individual primary studies.

The review is also constrained by its eligibility criteria. The explicit exclusion of non-English articles, non-peer-reviewed publications, and quantitative studies may introduce language and publication biases, potentially omitting relevant cross-cultural data.

## Conclusions

This systematic review identified key determinants of PA among candidates for and patients after MBS, synthesizing findings from 16 studies into 4 overarching thematic units: internal psychological, internal physical, external social, and external environmental factors. Internal physical barriers, particularly pain and functional limitations, were the most frequently reported obstacles, whereas internal psychological factors, including behavioral strategies and positive PA-related associations, emerged as the primary facilitators. These findings reaffirm the central role of physical and psychosocial elements in PA adaptation and highlight the need for interventions that integrate individualized and socially supported approaches. This review provides the first comprehensive synthesis of PA barriers and facilitators in the population of patients undergoing bariatric surgery and offers a foundation for developing targeted clinical recommendations.

## Supplementary Information

Below is the link to the electronic supplementary material.

Supplementary Material 1

## Data Availability

All data supporting the findings of this study are available within the paper and its Supplementary Information.

## Abbreviations

BMI: body mass index
MBS: metabolic bariatric surgery
PA: physical activity
POST: after surgery
PRE: before surgery
