# Supplementary material for: Perceived Barriers and Facilitators to Physical Activity in Patients Undergoing Bariatric Surgery: A Systematic Review of Qualitative Studies
Source: Obes Surg. 2026 May 21;36(7):3870–82. doi: 10.1007/s11695-026-08735-w (PMC13323194; doi:10.1007/s11695-026-08735-w)
Supplement: Supplementary file 1 — Supplementary Material 1 [file 11695_2026_8735_MOESM1_ESM.docx]

# **Supplementary Material**

Table S 1 Eligibility criteria

| **Criterion** | **Inclusion criteria** | **Exclusion criteria** |
| --- | --- | --- |
| *Population* | - Human individuals - Stp. any kind of MBS - With an indication for MBS | - People with obesity who did not underwent MBS and without an indication for MBS |
| *Topic* | - Any kind of PA - Factors influencing PA – barriers and facilitators | - General postoperative management - Nutritional management - Psychological management - Support groups |
| *Language* | - English | - A language other than English |
| *Date of publication* | - < August 1^st^, 2025 | - > July 31^st^, 2025 |
| *Article* | - Qualitative methodologies - Peer reviewed articles | - Exclusively quantititive methodologies - Case studies - Protocols - Thesis - Abstracts - Conference proceedings |

Table S 2 Search formulas

| **Databasis** | **Search formula** | **Additional information** |
| --- | --- | --- |
| PubMed | ((bariatric surgery OR bariatric*) AND (exercise OR sport OR physical activity OR training OR walk* OR lifestyle recommendation OR lifestyle management) AND qualitative) | Advanced search |
| PubMed MeSH | ("Bariatrics"[Mesh] AND ("Sports"[Mesh] OR "Exercise"[Mesh]) AND "Qualitative Research"[Mesh]) | - |
| Web of Science | ALL=(((bariatric surgery OR bariatric) AND (exercise OR sport OR physical activity OR training OR walk OR lifestyle recommendation OR lifestyle management) AND qualitative)) | Advanced search |
| Web of Science MeSH | Not available | - |
| EBSCO | ((bariatric surgery OR bariatric*) AND (exercise OR sport OR physical activity OR training OR walk* OR lifestyle recommendation OR lifestyle management) AND qualitative) | Advanced search; Medline is part of the EBSCO databasis |
| Medline MeSH | ((MH "Bariatrics") AND ((MH "Sports") OR (MH "Exercise")) AND (MH "Qualitative Research")) | - |
| Scopus | ("bariatric surgery" OR "bariatric*") AND ("exercise" OR "sport" OR "physical activity" OR "training" OR "walk*" OR "lifestyle recommendation" OR "lifestyle management") AND "qualitative"SCOPUS | Advanced search; limited to title, abstract, key words |
| Scopus MeSH | Not available | - |

Table S 3 Quality assessment using CASP qualitative checklist


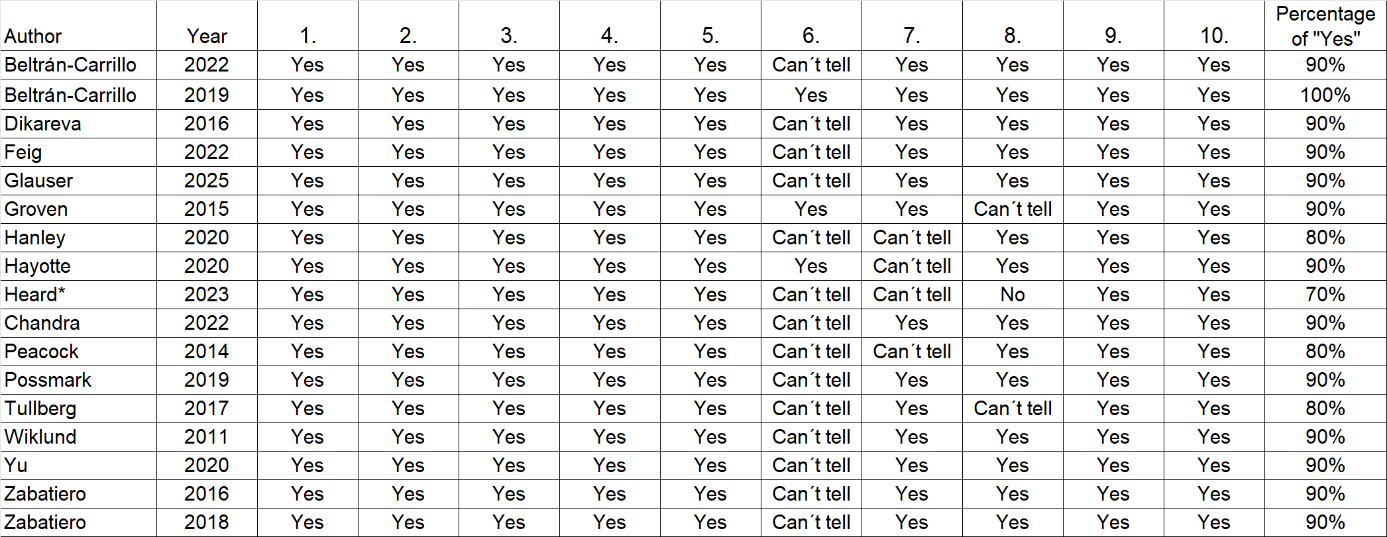
 1. Was there a clear statement of the aims of the research?

2. Is a qualitative methodology appropriate?

3. Was the research design appropriate to address the aims of the research?

4. Was the recruitment strategy appropriate to the aims of the research?

5. Was the data collected in a way that addressed the research issue?

6. Has the relationship between researcher and participants been adequately considered?

7. Have ethical issues been taken into consideration?

8. Was the data analysis sufficiently rigorous?

9. Is there a clear statement of findings?

10. How valuable is the research?**

*This reference is not included in the reference list because it does not appear in the main text.

Heard, A. L. (2023). *How Do Postoperative Bariatric Patients Stay on Task with Nutrition Goals and Physical Activity to Prevent Weight Regain? : Bariatric Times*. <https://bariatrictimes.com/postoperative-bariatric-nutrition-physical-activity/>

** Question 10 was evaluated focusing on three criteria: *1) whether the researcher discusses the contribution the study makes to existing knowledge or understanding (e.g., do they consider the findings in relation to current practice or policy, or relevant research-based literature; 2) whether they identify new areas where research is necessary; 3) whether the researchers have discussed whether or how the findings can be transferred to other populations or considered other ways the research may be used.* If the answer was „Yes“ for at least 2 criterias, the overall rating for Question 10 was also „Yes“. If the answer was „No“ for 2 or more criteria, the overall rating for Question 10 was „No“. If there was insufficient information, the for Question 10 was „Can´t tell“.

Table S 4 Barriers

„PA“ = physical activity

Table S 5 Facilitators

„PA“ = physical activity
